# Implying social interaction and its influence on gaze behavior to the eyes

**DOI:** 10.1371/journal.pone.0229203

**Published:** 2020-02-24

**Authors:** Gijs A. Holleman, Roy S. Hessels, Chantal Kemner, Ignace T. C. Hooge

**Affiliations:** 1 Experimental psychology, Helmholtz Institute, Utrecht University, Utrecht, the Netherlands; 2 Developmental psychology, Utrecht University, Utrecht, the Netherlands; 3 Brain Center, University Medical Center Utrecht, Utrecht, the Netherlands; SUNY Polytechnic Institute, UNITED STATES

## Abstract

Researchers have increasingly focused on how the potential for social interaction modulates basic processes of visual attention and gaze behavior. In this study, we investigated why people may experience social interaction and what factors contributed to their subjective experience. We furthermore investigated whether implying social interaction modulated gaze behavior to people’s faces, specifically the eyes. To imply the potential for interaction, participants received either one of two instructions: 1) they would be presented with a person via a ‘live’ video-feed, or 2) they would be presented with a pre-recorded video clip of a person. Prior to the presentation, a confederate walked into a separate room to suggest to participants that (s)he was being positioned behind a webcam. In fact, all participants were presented with a pre-recorded clip. During the presentation, we measured participants’ gaze behavior with an eye tracker, and after the presentation, participants were asked whether they believed that the confederate was ‘live’ or not, and, why they thought so. Participants varied greatly in their judgements about whether the confederate was ‘live’ or not. Analyses of gaze behavior revealed that a large subset of participants who received the live-instruction gazed less at the eyes of confederates compared with participants who received the pre-recorded-instruction. However, for both the live-instruction group and the pre-recorded instruction group, another subset of participants gazed predominantly at the eyes. The current findings may contribute to the development of experimental designs aimed to capture the interactive aspects of social cognition and visual attention.

## Introduction

Since the pioneering studies of Buswell [1] and Yarbus [2], many eye-tracking studies have shown that people have a strong tendency to look at other people’s faces, especially their eyes [3–7]. While previous studies have predominantly investigated how observers look at images and videos of people’s faces (for a discussion, see [8]), such ‘non-responsive’ stimuli may not adequately represent how people look at each other in the context of everyday social situations (for a review, see [9]). As Risko, Richardson & Kingstone [10] noted, many laboratory experiments uphold an *“illusory barrier between participants and the stimuli they are presented with…participants are not expected to interact with the faces presented to them or worry about what the stimuli might think of them”* (p. 70). In recent years, researchers in the field of social attention have increasingly focused on how visual attention and gaze behavior are modulated by the social context, for example, by the presence of ‘live’ people and potential interactions [9, 11, 12]. The study of gaze behavior in the context of social interaction is important to better understand, for example, how and when gaze behavior may support the pickup of cognitive and emotional states [13–15], how gaze facilitates social learning and development [16–18], and how gaze supports and regulates speech and turn-taking behavior in conversations [19–22]. Furthermore, understanding gaze behavior in the context of social interaction has several key implications for clinical research. For example, individuals scoring high on psychopathological traits of Autism Spectrum Disorder (ASD) and Social Anxiety Disorder (SAD) may display more ‘atypical’ gaze behavior during social interaction, such as gaze avoidance and a lack of ‘eye contact’ [23, 24]. The current challenge for researchers in the field of social attention is to design experiments that capture the relevant aspects of gaze behavior in social situations, such as the presence of other people and the possibility to interact with them, while also maintaining a high level of experimental control [25, 26]. In the present study, we investigated why people may experience social interaction and what factors contributed to their subjective experience. We furthermore investigated whether implying social interaction modulated gaze behavior to faces, specifically to the eyes. We will first discuss previous eye-tracking research on how the presence of other people and the potential for interaction modulates basic processes of visual attention, and then we will outline the research questions and hypotheses of the present study.

### Gaze behavior in context: Social presence and potential interactions

In the last decade, several eye-tracking studies have shown that people look differently at ‘live’ people as compared with images and videos of people [see 11, 12, 22, 27]. Two prominent examples are given by Foulsham et al. [27] and Laidlaw et al. [11]. Foulsham et al. [27] investigated where participants looked while walking on a university campus. The experimenters compared participants’ gaze behavior during their walk on campus with participants’ gaze behavior while watching the first-person video-recordings of themselves and others walking on campus. Foulsham et al. [27] discovered that when participants walked on campus, they were more likely to look away from other pedestrians on the street, especially when they approached upcoming pedestrians. Interestingly, when participants were presented with the first-person video-recordings, they continued to look at the pedestrians. Likewise, Laidlaw et al. demonstrated that participants looked less often and for shorter durations at a confederate sitting in a chair across the room in comparison with a confederate displayed on a computer screen in the same room.

The studies by Foulsham et al. [27] and Laidlaw et al. [11] both demonstrated that the tendency to look at other people, often assumed to be largely automatic and reflexive (for example, see [3]), was markedly less when other people were physically present. These findings converge on the notion that the presence of other people can cause substantial changes in a wide variety of behaviors, feelings, physiological states, motives, and beliefs. Such changes in cognition and behavior are sometimes referred to as ‘social presence’ or ‘mere presence’ effects [28–31]. Similarly, other eye-tracking studies have further demonstrated that gaze behavior can be modulated by social presence and potential interactions [9, 12, 32, 33]. However, it is not always clear under what circumstances social presence may cause changes in people’s gaze behavior, or how gaze behavior may be modulated as a result. For example, Freeth et al. [12] showed that when an interviewer engaged in ‘eye contact’ with interviewees, the participants who were presented with a ‘live’ interviewer gazed longer at the interviewer’s face (i.e. when the interviewer was physically present) in comparison with participants who were presented with an interviewer displayed on a computer screen. In this interview-context, looking at the eyes and face of the other person may be necessary to adequately monitor and respond to the interviewer (see also [22, 34]). However, in a waiting-room context, or while walking on campus (as in the experiments by Laidlaw et al. [11] and Foulsham et al. [27]), people may be more likely to avoid looking at other people in their physical proximity. In other words, different social and behavioral contexts, characterized by different social and behavioral norms, may produce particular patterns of gaze behavior associated with those contexts. For example, a phenomenon known as ‘civil inattention’ [35] describes how people often ignore each other in public places, for example, in waiting-rooms, elevators, and on public transportation. In these situations, people may briefly glance at each other to ‘acknowledge’ each other’s presence but consequently look away and politely ‘ignore’ each other (see also [36]). Conversely, in some other situations it may be considered inappropriate not to look at and respond to other people. For example, it would be strange not to actively attend to the other person at a job interview or when having coffee with a friend. In other words, certain social contexts may afford interaction or not [37], which likely influences how gaze behavior is allocated towards other people.

### Implied social presence—The belief of being watched

Researchers have also discovered that certain beliefs and attitudes about the presence of others influences visual attention and gaze behavior, for example, when people believe they are being watched or observed by others [38]. Several studies have shown that the implication of someone’s presence may already cause changes in how people allocate their gaze, for instance when people think they are being filmed [32, 39, 40]. In a study by Gobel et al. [39], participants were presented with several video clips of people’s faces under different viewing conditions. For half the videos, participants were instructed that they would simply look at the video clips, while for the other half, they were instructed that a webcam would also be recording them while watching the video clips. In the latter condition, Gobel et al. [39] told participants that their recordings would later be watched back by the people displayed on screen. Gobel et al. also added a description of the social status of the people in the video clips. Interestingly, they discovered that when participants believed they were being filmed by a webcam, they looked less at the eyes of high-ranking targets, but not of the low-ranking targets. In this study, Gobel et al. posited that gaze behavior to the eyes may be indicative of social rank signaling (see also [41]), similar to how some primate species and other animals visually communicate dominance and social status [42, 43]. Studies in primatology have demonstrated that lower-ranking members of the social hierarchy often look at the dominant members of the group, except when the high-ranking individuals gaze directly back at them [44]. However, Gobel et al. only prospectively implied social presence, meaning that participants were only told they would be evaluated by another person at a later point in time.

A more direct way of simulating social presence was achieved by Gregory & Antolin [45]. In this study, participants were led to believe that another person could see them via a so-called ‘live’ video-feed which was in fact a ‘staged’ video-feed. To investigate whether participants’ beliefs about the possibility for social interaction modulated gaze behavior to faces, participants could receive one of three instructions prior to viewing: (1) they would be presented with a ‘live’ video-feed of a person with the possibility to interact; (2) they would be presented with a ‘live’ video-feed of a person without the possibility to interact; or (3) they would be presented with a video clip of a person. Crucially, all participants were shown a pre-recorded video clip of a confederate. The video clip displayed a person who sat in a chair and looked at a clipboard. At one point, the confederate turns her head and looks directly into the camera for approximately four seconds. Gregory & Antolin [45] showed that when participants were instructed that the person displayed on the computer screen was ‘live’ and could potentially interact with them, participants gazed less at the person’s face in comparison with participants who were told that the presentation was a pre-recorded clip. Gregory & Antolin [45] also reported that when the person in the clip directly looked at the participant, which could be perceived as conveying the initiative to interact, participants were more likely to look at the face of the confederate regardless of the instructions received prior to the experiment. Gregory & Antolin [45] posited that: *“Had the confederate prolonged this period of direct gaze*, *overall gaze avoidance might have been evident as the participants attempted to reduce the “social risk” which results from making direct gaze with a stranger”* (p. 11). In other words, a prolonged period of direct gaze might result in overall gaze avoidance, because prolonged eye contact with strangers may be considered inappropriate or perceived as aggressive and threatening [41, 46–48].

Importantly, Gregory & Antolin [45] demonstrated that participants can be led to believe that there is a potential to interact with a ‘live’ person displayed on a computer screen, even though this person was only displayed as a video clip. However, the video clip of the confederate in this study may have only minimally conveyed a potential to interact, which may have reduced the experience of being watched by another person, as well as the experience of potential interaction. Gregory & Antolin [45] also reported that many participants in this study did not believe that the confederate was ‘live’, or that the confederate could really interact with them (as reported through several questionnaire items after the experiment). But why did some participants believe that they were looking at a ‘live’ person and others did not? And why did some participants believe there was a possibility for interaction and others did not? Previous studies have not always explicitly focused on why people may experience social interaction or why they perceive a potential for social interaction.

### Present study

In the present study, we wondered why people may experience a potential for social interaction and what factors may contribute to this experience? To investigate this, we specifically wanted to imply social *interaction* instead of social *presence* [39, 45]. Also, we investigated whether implying social interaction would also modulate gaze behavior to the eyes. We reasoned that staging a face-to-face encounter with a stranger would be the most suitable context for our research question. We wanted to create a situation in which a person appears to ‘look’ directly at the participant, and thereby minimize the potential ambiguity as to whether that person is actively attending the participant. Based on previous research, one might expect that if someone believes that another person can directly see them, some people may avoid that person’s gaze. The ‘social risk’-hypothesis, for example, posits that a prolonged period of direct gaze from a stranger results in gaze avoidance [45]. We devised an experiment to investigate whether people also avoid someone’s gaze if they believe they are being watched by a ‘live’ stranger and, importantly, when they believe there is a potential for social interaction. In our study, participants were presented with video clips of confederates who displayed more ‘interactive’ behavior (e.g. several bids for social interaction) compared with the behavior exhibited by confederates in previous studies (cf. [11, 39, 45]). Prior to every presentation, one of four confederates walked into a separate room in order to suggest to participants that they were taking place behind a webcam. After the confederate entered the separate room, some participants were instructed that they would be presented with the confederate via a ‘live-video connection’ and some participants were instructed that they would be presented with a pre-recorded clip of the confederate. In fact, regardless of the instructions, all participants were shown a pre-recorded video clip. During the presentation, we recorded participants’ eye movements with a high-end eye-tracker. This allowed us to measure gaze behavior to facial features with high temporal and spatial resolution. Afterwards, participants were asked whether they believed that they were presented with a ‘live’ person and why they thought so.

## Materials and methods

### Participants

Eighty-two participants (fifty females; *M*_*age*_ = 31.69 years, range 18–70, *SD* = 11.66) were recruited amongst visitors of the “Betweter Festival”, which is an annual science festival organized in Tivoli Vredenburg, a large concert venue in Utrecht. Based on previous studies [39, 45], we aimed to recruit a minimum of sixty participants. We reasoned this would be sufficient for our research questions and hypotheses. To compare, Gregory & Antolin’s study used a similar sample size to compare a pre-recorded-viewing group (28 participants) and a ‘two-way’-viewing group (34 participants). All participants reported normal or corrected-to-normal vision. The experiment was conducted in a large room where other activities were also going on. The room was noisy with moderately loud music and people.

### Ethical considerations and informed consent

This study was approved by the Ethics Committee of the Faculty of Social and Behavioral Sciences of Utrecht University and is registered under protocol number FETC17-097. Written informed consent was obtained from each participant prior to the start of the experiment. The confederates who participated in this study also gave written informed consent for the publication of the stimulus video clips and illustrations of this material.

### Stimuli and apparatus

**Fig 1:**
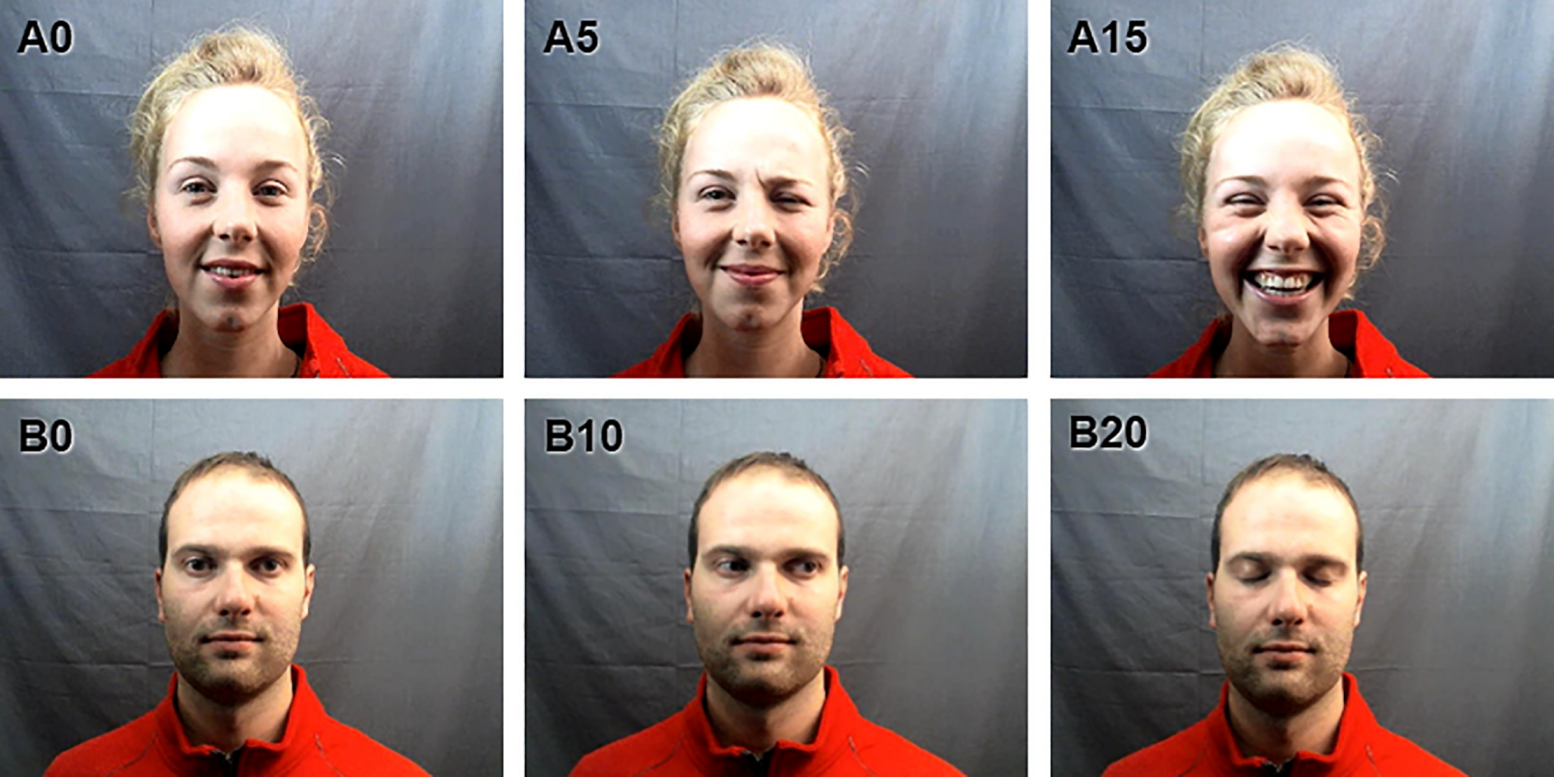
Snapshots of video clips. In video clip A (upper panels), the confederate first looks directly into the camera (**A0**). After 5 seconds, the confederate frowns and smiles and then resumes looking into the camera (**A5**). After 15 seconds, the confederate laughs and then resumes looking into the camera again (**A15**). Finally, after 25 seconds the confederate winks and smiles. In video clip B (lower panels), the confederate first looks directly into the camera with a neutral expression (**B0**). After 10 seconds (**B10**), the confederate averts his gaze to the right for about 5 seconds. Then, after 20 seconds, the confederate briefly closes his/her eyes and then looks back into the camera again (**B20**).

We aimed to imply social interaction via a staged ‘face-to-face’ interaction, albeit mediated through a computer screen. To achieve this, we recorded stimulus material in a two-way video setup devised by Hessels et al. [49]. This setup allowed us to create video clips of the confederates as if they were looking directly at each other while simultaneously looking into the camera. Similar recording techniques have previously been used by cinematographers to achieve a more engaging performance (e.g. actors, interviewees, models). We reasoned this would then create a more engaging viewing experience for the participants, as compared with watching a recording of a person who models in front a camera lens. We used four confederates as models (2 females, 2 males), and recorded two video clips of each confederate. Each clip displayed a frontal view of the confederate who exhibited various facial expressions which were meant to exhibit ‘responsive’ and ‘interactive’ behavior. We labeled the clips ‘video clip A’ and ‘video clip B’, respectively. Video clip A displays a confederate who looks directly into the camera, frowns, smiles, laughs, and then winks. Video clip B displays a confederate who first looks directly into the camera with a neutral expression, averts his/her gaze for a couple of seconds, and then shifts back to direct gaze. Finally, the confederate closes and opens his/her eyes for a second. The sequence of these events was the same for every confederate with slight variations in timing (see Fig 1 for snapshots from the video clips). Each video clip lasted approximately 30 seconds and was recorded with a Logitech webcam recording video frames of 800 by 600 pixels at 30Hz. The video frames were scaled to a resolution of 1024 by 768 pixels during the presentation. The rationale behind these social gaze cues and facial expressions was partly based on the literature. Direct gaze (‘eye contact’) and averted gaze are well documented as powerful social signals [48]. Furthermore, we reasoned that smiling, frowning, laughing and winking would be appropriate to convey ‘interactive’ behavior within the context of this experiment. However, note that the goal of this study was not to investigate the influence of particular social gaze cues on gaze behavior, but rather to create some variation in the behavior of confederates to imply social interaction. Also, we did not include speech or audio in our video-recordings.

**Fig 2:**
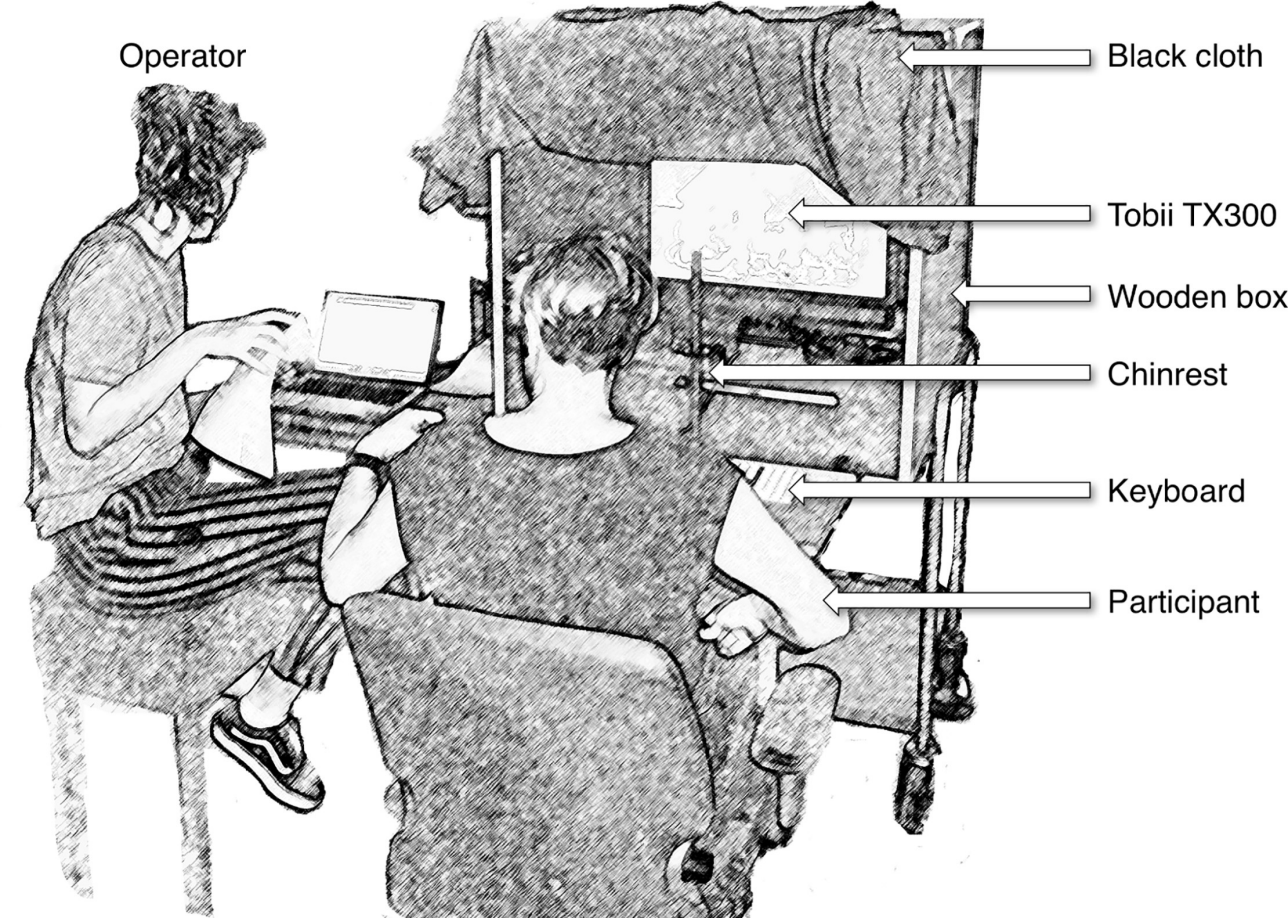
The eye-tracking setup consisted of a box placed on a cart. The box contained a Tobii TX300 eye tracker and presentation screen. A small desk with a keyboard was attached to the front of the box and a chinrest was mounted unto the front of the desk. In this illustration, the participant receives instructions from the operator. When the operator was ready, the participant would take place in the chinrest and the black cloth was draped over the participant. Reprinted from [53] with permission.

To record participants’ eye movements, a Tobii TX300 eye tracker with an integrated 23-inch monitor (1920 by 1080 pixels; 60 Hz refresh rate) was used (Tobii Technology, Stockholm, Sweden). The Tobii eye-tracking system ran at 300 Hz and communicated with MATLAB (version R2012b, MathWorks Inc., Natick, MA, USA) running on a MacBook Pro (2.8 GHz i7 processor, OS X 10.9) via the Tobii SDK (*Tobii Pro Analytics SDK*, *version 3*.*0*). Stimulus presentation was done using the PsychToolbox (version 3.0.11; [50]). The operator controlled the stimulus computer and eye tracker. The eye tracker was placed inside a box with a black cloth attached to the top and sides of the box (see Fig 2). The eye tracker was located at 65 cm from the participant. The video clips were centered on the screen of the Tobii and subtended 23.6° by 17.8° under the assumption that the viewing distance was approximately 62 cm. A chin- and forehead rest was used to keep the viewing conditions constant for every participant, minimize head movements, and ensure optimal data quality [51, 52].

### General procedure

**Fig 3:**
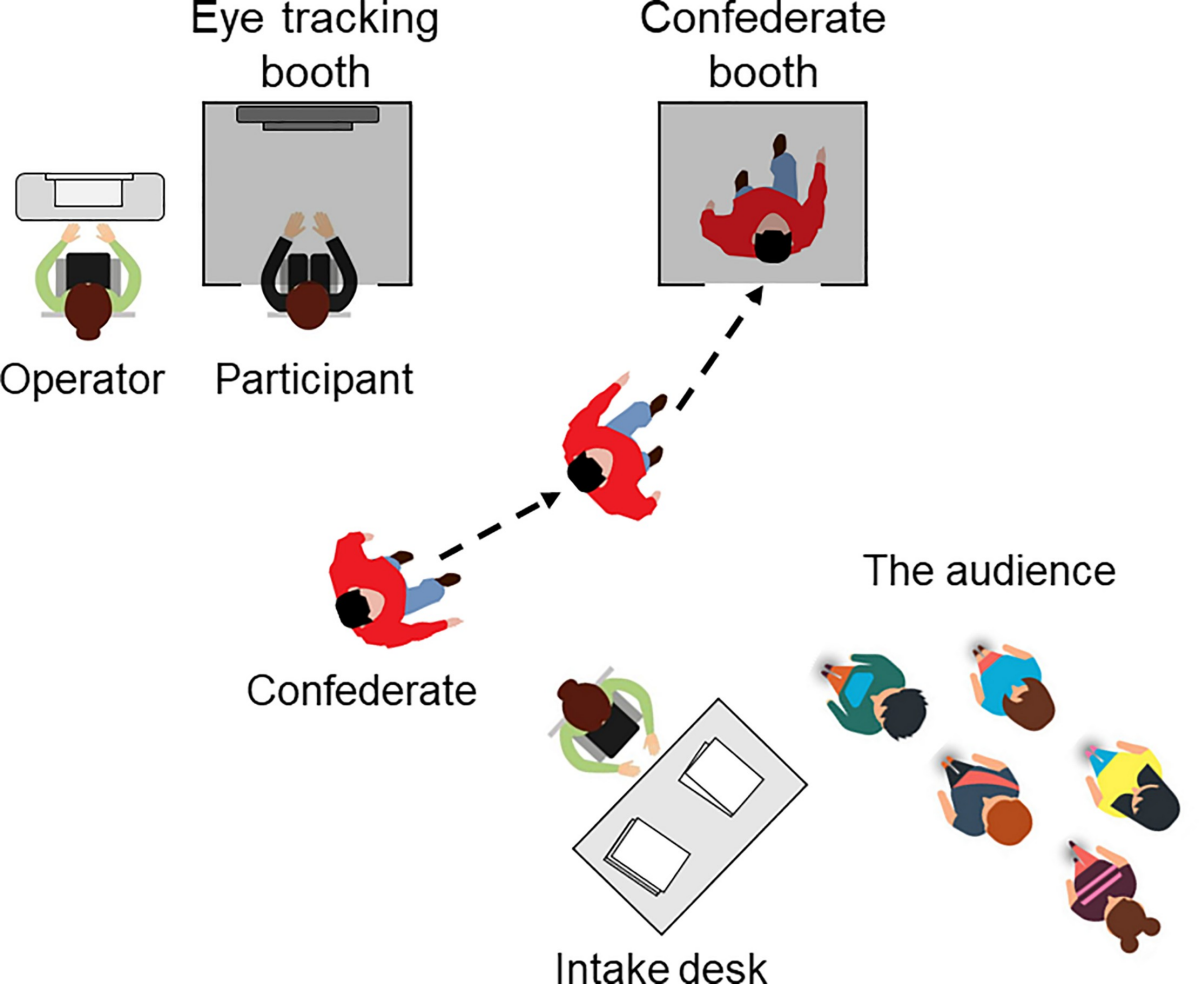
Schematic top-view of the research setting. The participant is seated in the eye-tracking booth next to the operator. The confederate (dressed in a red sweater) walks into the confederate booth after being called for by the operator prior to each measurement. A queue of participants (the audience) is awaiting their turn after they signed up to participate at the intake desk. Note that this illustration is a simplified view. During the actual experiment, three more confederates dressed in red sweaters were walking around and the research setting was surrounded by other people, a bar, lights, as well as other installations and performances.

Participants were recruited at the festival site. The research setting consisted of an intake desk where visitors of the festival could sign up for participation. The experiment was promoted as follows: *“This experiment is about social interaction*. *We are not allowed to say more about it*. *Do you dare to step into this black box*?*”* Prior to the experiment, participants were given a brief information letter. Participants gave written informed consent and provided some basic demographic information. Every participant received a unique participant code which was coupled with a specific presentation procedure (see below). After participants signed up for the experiment, they were asked to line up in a queue and await their turn. At the research setting, the four confederates walked around in bright red sweaters to signal their participation in the experiment. Visitors and participants could talk to the confederates prior and after the experiment, but the confederates did not discuss the content of the experiment. At the start of every experiment, the operator asked the first participant in line to come forward to be positioned in the eye-tracking booth. The operator then entered the unique code of the participant into a custom MATLAB script. Based on the participant code, every participant was coupled to one of the four confederates, combined with a specific instruction-type and video clip-type. This was done to ensure that we would have an approximately equal distribution of participants over the different confederates, instructions, and video clips at the end of the testing session. After the participant was positioned, the operator loudly asked the confederate assigned to the participant to get ready. The confederate then responded in an affirming way and walked past the participant and the other people present at the scene to step into the confederate booth (see Fig 3). The confederate’s booth consisted of a wooden cubicle with a black curtain and was placed next to the eye-tracking booth. The confederate’s booth was placed so that the current participant, as well as the other people waiting in line for their turn to participate (the audience), would be able to see the confederates walking in and out of the booth before and after the experiment.

#### Calibration procedure

After the participant was positioned in front of the eye tracker and the confederate had stepped into the confederate’s booth, the operator instructed the participant how to control the self-paced calibration procedure by using a keyboard press. The operator then draped a black cloth over the participant’s head. Participants then conducted a self-paced 9-point calibration procedure (participant-controlled calibration has been shown to result in better accuracy and precision than operator- or system-controlled calibration, see [54]). After the calibration procedure, the operator visually assessed the calibration output. In some cases, the calibration output yielded calibration points either without any data, or with specific inconsistencies (e.g. dispersed gaze points around the calibration point). If necessary, these points were re-calibrated. After examining the calibration output of the Tobii SDK, and after recalibration if this was necessary, the operator instructed the participant to initiate the experiment by a spacebar press.

#### Instructions to participants

Approximately half of the participants received the *live-instruction* and the other half received the *pre-recorded-instruction*. Based on the unique participant code, participants received one of the instruction-types. The instruction appeared on the computer screen after the participant initiated the start of the experiment by a spacebar press (directly after the calibration procedure).

The *live-instruction* stated: *“You are about to look at [name confederate] through a live-video connection*. *[Name confederate] can see you too via the webcam*. *There is no audio-connection*, *so you cannot hear each other*. *The experiment lasts approximately one minute*. *Press the spacebar to start”*.

The *pre-recorded-instruction* stated: *“You are going to look at a video clip of [name confederate]*. *The video clip is pre-recorded*. *The experiment lasts approximately one minute*. *Press the spacebar to start”*.

After the participant read the instruction and pressed the space bar, the presentation started directly. Note that both the instructions did not explicitly task the participant to look at the face of the confederate or otherwise to behave in a certain way. Instead, the instructions were formulated in this way to set up the conditions of the experiment, namely to imply to participants whether the presentation of the confederate was ‘live’ or not and whether social interaction was possible.

#### Presentation procedure

Based on their participant code, participants could either receive video clip A or video clip B (see Fig 1) of the confederate to whom they were assigned. Each video clip presentation lasted approximately 30 seconds. When the video clip ended, a calibration-validation procedure was conducted. This part was included to assess the accuracy of the eye-tracking data. Hereafter, a final screen appeared which stated: *“End of the experiment”*. The participant then left the eye-tracking booth and was directed towards a desk by the operator. Here, the participant was asked to fill in a short questionnaire about their subjective experience during the experiment.

#### Questionnaire and debriefing after the experiment

The questionnaire at the end of the experiment contained the following text and questions: *“You have just seen [name confederate]*. *In this experiment*, *some people are shown a person via a live-video stream*, *and some people are shown a pre-recorded video of a person*. *However*, *the instructions you received prior to the experiment do not necessarily have to correspond to what you have seen*, *but it is possible*. *Now*, *do you think that you were looking at [name confederate] via a live-video connection*, *or that you have looked at a pre-recorded video clip*? *Tick the box to answer*. *(1) a live-video connection*, *or (2) a pre-recorded video clip”*. This was done to investigate whether participants believed whether the confederate was ‘live’ or not, regardless of the instructions they received prior to the presentation. Hereafter, participants were asked: *“How confident are you about your judgement*? *Please rate how confident you are by putting a cross on the confidence-scale*. *0 means that you are completely not confident and 10 means you are completely confident”*. This questionnaire item consisted of a 10-point scale. Finally, the questionnaire contained one more open question asking participants to elaborate on why they chose their answers to the previous questions. After filling in the questionnaire the participant was dismissed. We did not debrief the participants directly after the experiment, because some participants might have talked to other visitors (and potential participants) about the experiment. Instead, we posted a general debriefing on the festival’s public webpage in which we revealed the setup of the experiment, as well as some preliminary results about how many people believed that the presentation was ‘live’ or not.

### Eye-tracking data: Signal processing and analysis

Eye-tracking data of the left eye were imported into MATLAB 2017a. Subsequently, we used custom MATLAB software to process the data according to the following steps:

1. **From raw data to fixations.** As a first analysis step, we classified fixations with a modified version of the adaptive velocity-threshold method devised by Hooge & Camps [55]. We chose this algorithm because it can classify fixations on slowly moving objects (a moving face in our case). To produce a velocity signal, we fitted data segments of seven subsequent data points in the position signal with a parabola (instead of three as in [55]). The derivative of the fitted function was used to assign a velocity to the center-point (the fourth of the seven data points). This was done for all data points except the first and the last three of the eye-tracking data. The absolute velocity signal was computed by taking the magnitude of the combined horizontal and vertical eye-tracking signal. All data points having absolute velocities higher than the threshold (the average velocity plus three times the standard deviation of the velocity) were removed. This procedure was repeated until the velocity threshold converged to a constant value or until the number of repetitions reached 50. All episodes that survived the data removal were labeled as fixations. One may argue that we label smooth pursuit episodes as fixations because: 1) our visual stimuli contain slowly moving objects, and 2) our fixation classifier basically selects slow phases from the gaze-position signal containing both slow and fast phases. Note that this is intentional because in this study we define a fixation as a period during which a (moving) object in the world is projected to roughly the same location on the fovea (for a discussion on definitions of fixations, see [56]). The product of the fixation classification is a list with subsequent fixations and their properties (fixation start time, fixation duration and mean fixation position per fixation).
2. **Determining positions of facial features in the video.** Our area of interest (AOI) analysis is based on a recently developed technique [57], which employs open-source face-detection and facial-landmark detection algorithms [58, 59]. This method has been extensively validated for eye-tracking data of observers watching videos of faces [57]. In the current analysis, we used this technique to determine the position of the eyes, nose and mouth for every frame of the video clip.
3. **Assigning AOI labels to fixations.** For each fixation start, we determined the closest preceding video frame start in time. This video frame was linked to the fixation. For each frame, we have coordinates of the centers of facial features. To link a fixation to a facial feature (from now on called an AOI), we computed the distances from the fixation location to the centers of the facial features. The AOI label of the closest facial feature is assigned to the fixation. However, if this distance is larger than 120 pixels (3.05º) we labeled this fixation ‘Other’-AOI because the fixation is outside the eyes, nose and mouth AOI. This method has previously been described as the Limited-Radius Voronoi Tessellation (LRVT) method and has been extensively validated as an objective and noise-robust solution that allows for better between-group and cross-study comparisons of AOIs in face-scanning research (for more details on this method, see [60]). The AOI span in the current study was 2.035º, reflecting the *“mean distance from each AOI cell center to the cell center of its closest neighbor”* [60] (p. 1701).
4. **From fixations to dwells.** We transformed the fixations into ‘dwells’ by determining the uninterrupted visits in AOIs (see [61], pp. 189–190). From this, we also computed dwell time, that is the time from entering to leaving a specific AOI (eyes, nose, mouth). Note that the duration of saccades made within a specific AOI is included in the dwell time measure.
5. **Relative total dwell time.** Finally, we computed a relative total dwell time measure for each participant. This measure was computed by summing up the dwell times for each AOI (eyes, nose, mouth and ‘other’–AOI) and dividing them by the duration of the video clips. This was done because the video clips contained slight variations in timing. Thus, the relative total dwell time measure indicates what percentage of the total video-clip duration, approximately 30 seconds, a participant spent looking at one of the AOIs. For example, a person might spend 60% of the time looking at the eyes AOI, 20% looking at the nose AOI, 15% at the mouth AOI, and the remaining 5% at the ‘other’-AOI. The relative total dwell time measure will be used in all subsequent analyses to assess how much a person looked at one of the areas of the face.

## Results

### Did participants believe there was a ‘live’ person or not?

Our first research question was to investigate why people experience a potential for social interaction and what factors contributed to this experience. Therefore, we first assessed whether participants believed there was a ‘live’ person or not. We analyzed participants’ responses to the questions given after the experiment. 1 person could not decide and was therefore excluded from the following analyses. Of the remaining 81 participants, 38 participants responded ‘live-video connection’ (46,9%) and 43 participants responded ‘pre-recorded clip’ (53,1%). Next, we tested whether the participants’ responses and the instructions they received prior to viewing the confederate were related to each other. In other words, we wanted to know whether instruction-type (‘live’ or ‘pre-recorded’) predicted how participants responded to the questionnaire items, even though all participants were told that the instructions did not necessarily correspond to the presentation. A chi-squared test conducted in JASP [62] showed that there was no significant association between participants’ responses and instruction-type (χ^2^ (1) = 3.466, *p* = 0.063) at an alpha level of 0.05. The observed phi-coefficient was 0.207, which is considered a small-to-moderate effect size [63, 64]. Although we could not show that instructions had a statistically significant effect on how participants responded, the instructions did seem to have some influence given the small-to-moderate effect size estimate. Moreover, looking at the proportions of participants whose responses aligned with the instruction-type, we observed that 57.8% of the participants who received the live-instruction answered ‘live-video connection’ and 62.8% of the participants who received the pre-recorded-instruction answered ‘pre-recorded clip’. Conversely, participants’ responses did not align with the received instruction-type. 42.2% of the participants who received the live-instruction answered ‘pre-recorded clip’ and 37.2% of the participants who received the pre-recorded-instruction answered ‘live-video connection’. As such, these results need to be interpreted with some caution. Next, we also tested whether participants' responses were dependent on the video-clip type (video clip A vs. video clip B). A chi-squared test showed there was no significant association between participants' responses and video clip-type (χ^2^ (1) = 0.302, *p* = 0.582) at an alpha level of 0.05. The observed phi-coefficient was 0.061. The effect size estimate was very small and thus it is not very likely that video clip-type modulated participants’ responses to this questionnaire item. In Table 1, we depict the proportion of participants who answered ‘live-video connection’ or ‘pre-recorded clip’, categorized by instruction-type and video clip-type.

**Table 1:** Participants’ responses (N = 81).

	Response	
Condition	Live-video connection	Pre-recorded clip
**Live instruction (N = 38)**	**27.2%**	**19.8%**
*Video clip A*	14.8%	8.7%
*Video clip B*	12.4%	11.1%
**Pre-recorded instruction (N = 43)**	**19.7%**	**33.3%**
*Video clip A*	7.4%	19.8%
*Video clip B*	12.3%	13.5%
**Total (N = 81)**	**46.9%**	**53.1%**

Finally, we tested whether participants' responses depended on instruction-type and video clip-type combined. In other words, we tested whether participants’ responses were a result of an interaction between instruction-type and video clip-type. A chi-squared test revealed that this was not the case (χ^2^ (3) = 5.674, *p* = 0.129). The effect size estimate was 0.265 (here computed as a Cramer’s *V* coefficient), which is considered a small-to-moderate effect size. Furthermore, participants who answered that the confederate was ‘live’ did not differ from those who answered ‘pre-recorded’ in their confidence ratings (measured on a scale from 1 to 10). Moreover, the confidence ratings did not substantially differ when we compared participants based on instruction-type and video clip-type.

### Why did participants believe the person was ‘live’ or not?

In order to further investigate why participants experienced a potential for interaction and what factors contributed to this experience, we also analyzed participants’ first-person responses to the open question after the experiment. Participants were asked to give a brief explanation as to why they answered that the person displayed on screen was ‘live’ or not. First-person responses were labeled and then grouped into several overarching categories by authors GAH and RSH. During this coding process, we found that some participants gave multiple reasons to support their answer. Therefore, we coded the responses separately for each reason provided. The full coding scheme is depicted in Table 2. Note that the reported numbers in Table 2 and in the text do not always refer to the number of participants, but instead to the number of responses given by participants that were coded under a specific label (these numbers are reported in *italics*).

**Table 2:** Coding scheme for first-person responses (N = 81).

Categories / labels	Response	
	Live video-connection	Pre-recorded clip
**Attitude (14)**	**6**	**8**
Instructions (8)	3	5
Improbable / skeptical (2)	0	2
Random / no reason provided (4)	3	1
**Behavior of confederate (44)**	**20**	**24**
Good timing behavior (14)	13	1
Strange timing behavior (4)	2	2
No cause behavior (12)	1	11
Authentic / natural (2)	2	0
Rehearsed / planned (11)	1	10
Experience of eye contact (1)	1	0
No experience of eye contact (2)	0	2
**Context (18)**	**12**	**6**
Match confederates (14) (e.g. clothing, talking, location)	12	2
Mismatch confederates (4) (e.g. appearance, location)	0	4
**Technicalities of setup (9)**	**2**	**7**
Image quality (2)	1	1
Image too light (3)	1	2
No camera visible (1)	0	1
Room mismatch (1)	0	1

1. **Attitudes.** In this category, we labeled answers that were suggestive of participants’ attitudes and skepticism regarding the experiment (*2*), or when participants did not provide any specific reasons (*4*). For example, one participant responded: *“During the experiment I already questioned whether it was ‘live’ and after this question I doubt it even more”*. Another participant answered: *“No idea*, *it can both be true”*. Participants in this category also referred explicitly to the instructions given prior to the experiment (*8*), even though participants were specifically told that the instructions did not necessarily correspond to whether the presentation was ‘live’ or not. For example, one participant who answered ‘pre-recorded clip’ responded: *“This was described in the instruction”*. In total, 6 participants whose answers were labeled under ‘Attitudes’ answered ‘live-video connection’ and 8 participants answered ‘pre-recorded clip’.
2. **Behavior of the confederate.** In this category, we labeled answers that explicitly referred to the confederate’s behavior. Many participants who believed that the confederate was ‘live’ answered that they perceived synchrony and reciprocity with the confederate’s behavior *(14)*. For example, these participants wrote: *“There was action-reaction”*, *“It felt very natural*, *like she was interacting with me”*, *“He reacted when I laughed”*, and *“It was like she was mirroring me”*. Interestingly, many participants who commented on the confederate’s behavior also answered ‘pre-recorded clip’. This group either answered that the behavior of the confederate appeared to have no particular cause *(12)*, or that they perceived ‘strange’ or ‘inappropriate’ timing in the behavior of the confederate (*4*). For example. one participant responded: *“She winked*, *and I winked back*, *but then she did not react as I expected”*. Similarly, other participants in this group wrote: *“His reaction / facial expression did not seem to have a cause”* and *“He smiled at a strange moment”*. Others also commented that the behavior of the confederate seemed rehearsed (*11*), for example, one of these participants answered: *“It looked fake*, *the way he moved his eyes looked planned and rehearsed”*. In total, 20 participants whose answers were labeled under ‘Behavior of Confederate’ answered ‘live-video connection’ and 24 participants answered ‘pre-recorded clip’.
3. **Context.** In this category, we labeled answers of participants who commented on specific aspects of the research context (*18*), such as the fact that confederates walked in and out of the booth, the confederates clothing, or that they were talking with confederates prior to the experiment. For example, one participant answered that: *“I saw the person walking into the booth”* and another participant responded: *“I just talked to him*, *and he was wearing the same shirt”*. 12 participants in this ‘Context’ category answered ‘live video-connection’ and 6 participants answered ‘pre-recorded clip’.
4. **Technicalities.** A small group of participants commented on several technicalities of the experimental setting (*9*). For example, one participant responded: *“It was bright in the room*, *not dark”*, mentioning the fact that the confederate booth from the outside seemed dark inside, while in the video the background of the room was illuminated. Furthermore, some participants commented on incongruencies and mismatches with the confederate or the room. For example, one participant wrote: *“He was sweating when I just saw him*, *but not in the video”*. From this category, 7 participants answered ‘pre-recorded clip’ and 2 participants answered ‘live video-connection’.

To conclude, we identified four main categories (see Table 2) under which we could label participants’ first-person responses. These factors seem to be important for participants’ experience of social interaction. The category: ‘Behavior of Confederate’ captured by far the largest part of participants’ responses, showing that how participants experienced the behavior of the confederate was strongly associated with whether the participants responded that the presentation was ‘live’ or a ‘pre-recorded clip’.

### Eye-tracking data quality assessment

**Fig 4:**
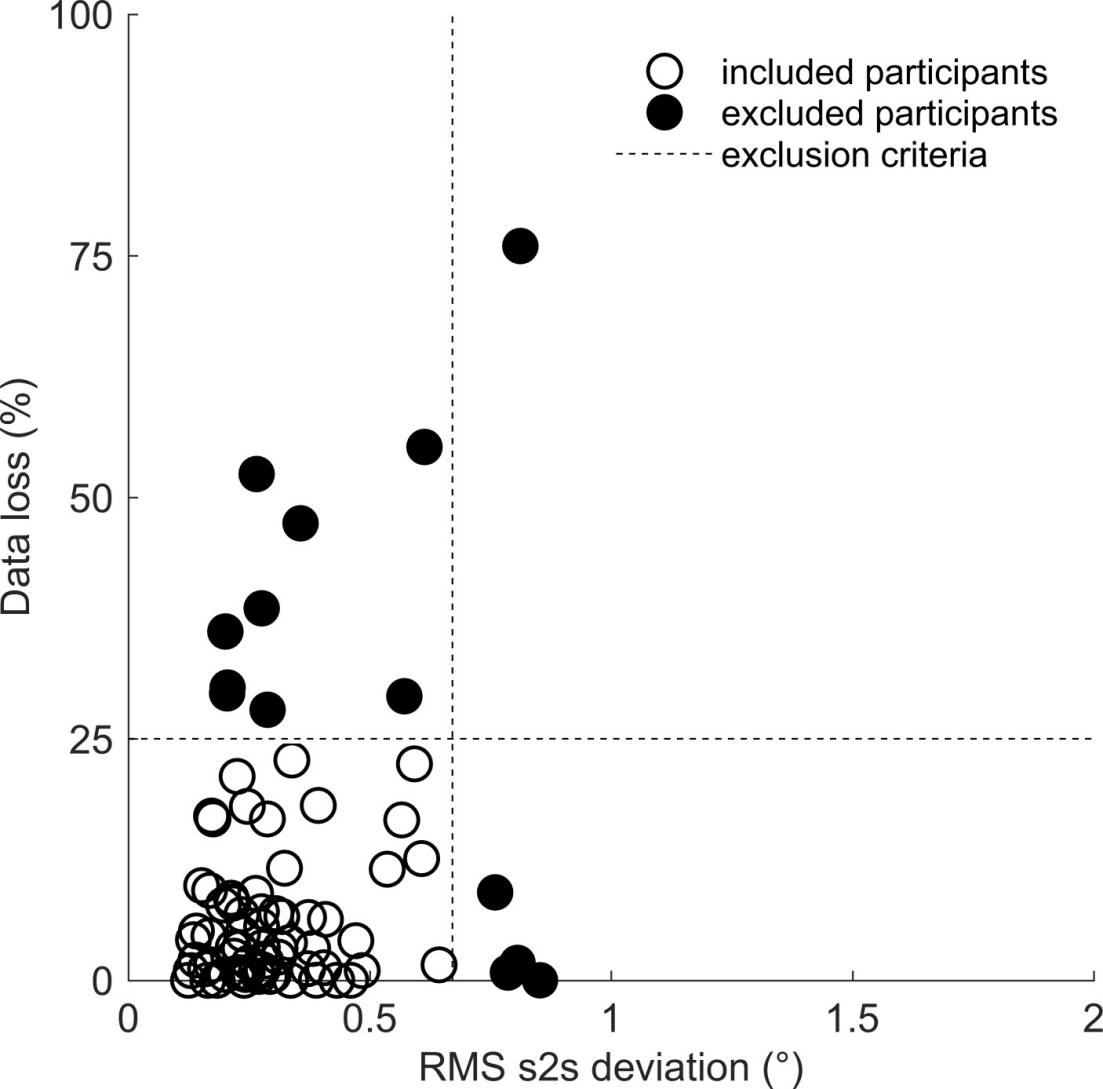
Visualization of data loss and RMS sample-to-sample (s2s) deviation. Every circle represents one participant. The dashed lines represent the exclusion criteria used; data loss > 25% on the y-axis, RMS s2s deviation (°) > 2 SD’s above the mean on the x-axis. Open circles represent the included participants (N = 68), closed circles represent the excluded participants (N = 14).

Prior to analyzing participants’ gaze behavior, we first assessed eye-tracking data quality. Data quality is an important part of eye-tracking research and essential to the validity of study results [54, 65]. The following measures were used. First, we computed the percentage of data loss per participant (i.e. no gaze coordinate was reported). Second, we determined precision by computing root mean squared sample-to-sample deviation (RMS s2s) of the raw gaze position signals for every participant [61]. If eye-tracking recordings showed more than 25% data loss, or if the precision of the recordings exceeded 2 SD’s above the mean RMS displacement, we excluded these recordings from subsequent analyses. Based on these criteria, 14 out of 82 participants were excluded (see Fig 4 for data loss, RMS s2s deviation and the exclusion criteria). Next, we computed the accuracy (i.e. systematic error) for the remaining 68 participants by measuring the difference between the average fixation location and the calibration point (averaged over participants and calibration points). The systematic error was found to be 0.65° on average (for more details, see [53]). We concluded that data quality for the remaining 68 participants was adequate for the subsequent analyses, because the average systematic error was approximately three times as low as the average AOI span of 2.035° (see Method section). Furthermore, data loss and RMS s2s deviation were low enough to allow for adequate fixation classification.

### Relative total dwell times on face AOIs

**Fig 5:**
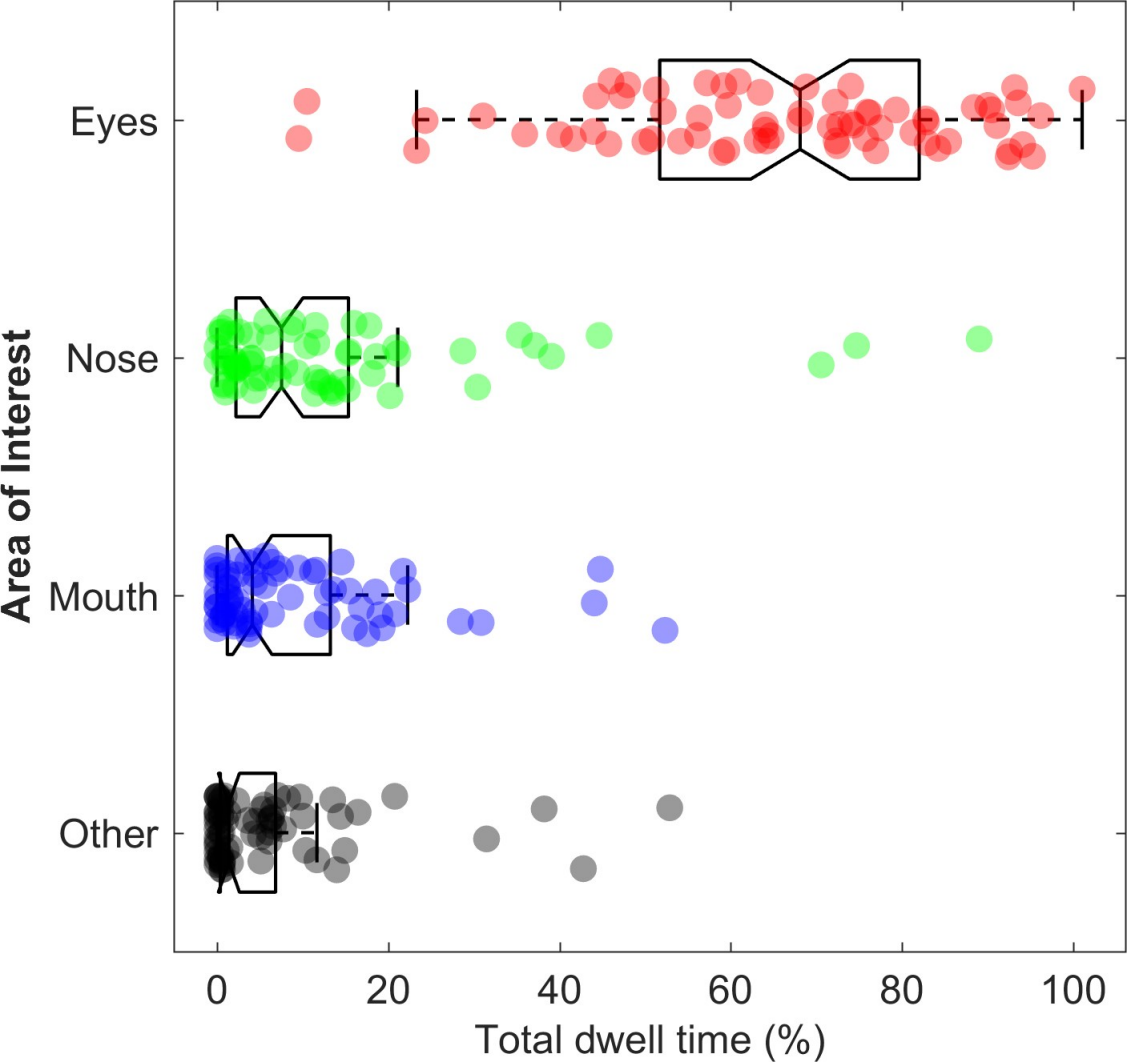
Box and whisker plots for relative total dwell time (%) per area of interest. Boxplots are overlaid with individual data points which are jittered in the vertical direction to avoid overlap. Each dot represents data from one of 68 participants. Boxes cover the 25^th^ to 75^th^ percentile (inter-quartile range; IQR). The middle of the box represents the median. Whiskers extend from the 25^th^ percentile and 75^th^ percentile to cover all data points lying within 1.5 times the IQR from the 25^th^ and 75^th^ percentile respectively. Notice that the relative total dwell times (%) on the nose, mouth, and ‘other-AOI are not normally distributed. Many participants gazed at these AOIs only for a relatively small amount of the video clip duration (≈ 30 seconds), or did not look at these AOIs at all, hence the bounded and skewed distributions and the large clusters of datapoints at or near zero.

As stated previously, we wanted to investigate whether implying social interaction modulates gaze behavior to the eyes. However, first we analyzed how participants looked at the face of the confederate irrespective instruction-type and video clip-type. It is clear from Fig 5 that many participants gazed predominantly at the eyes AOI. Furthermore, as shown by the individual data points, there is a wide range in relative total dwell time per AOI across individuals. Some participants gazed approximately 70–100% of the time at the eyes, while other participants gazed at the eyes for 10–50% of the time. Moreover, some participants gazed at the nose, mouth, or ‘other’–AOI for a relatively large amount of the time, while other participants only briefly looked at these AOIs, or not at all. Inter-individual variability in gaze behavior to facial features has been described in more detail in previous eye-tracking studies [66–69]. In short, these studies show that face scanning behavior can be quite idiosyncratic and remain stable over time. Furthermore, face scanning behavior is also likely influenced by particular task demands (e.g. face recognition, emotion recognition, free viewing).

### Does implied social interaction modulate gaze behavior to the eyes?

Our second research question was whether implying social interaction would modulate how people look at faces, specifically the eyes. As described previously, we expected that participants who believed that they were presented with a ‘live’ person would gaze less at the eye region of a face compared with participants who did not believe they were presented with a ‘live’ person. However, the first-person responses indicated that participants varied greatly in their beliefs about whether the confederate displayed on screen was ‘live’ or not and why they thought so. Therefore, we first analyzed relative total dwell time on the eyes AOI as a function of instruction-type. Subsequently, we analyzed relative total dwell time on the eyes AOI as a function of participants’ post-hoc beliefs. Note that participants who received the live-instruction are not necessarily the same as the participants who believed the confederate was ‘live’. The numbers of participants per group are reported for the specific analyses in the following sections.

#### Gaze behavior to the eyes as a function of instruction-type

**Fig 6:**
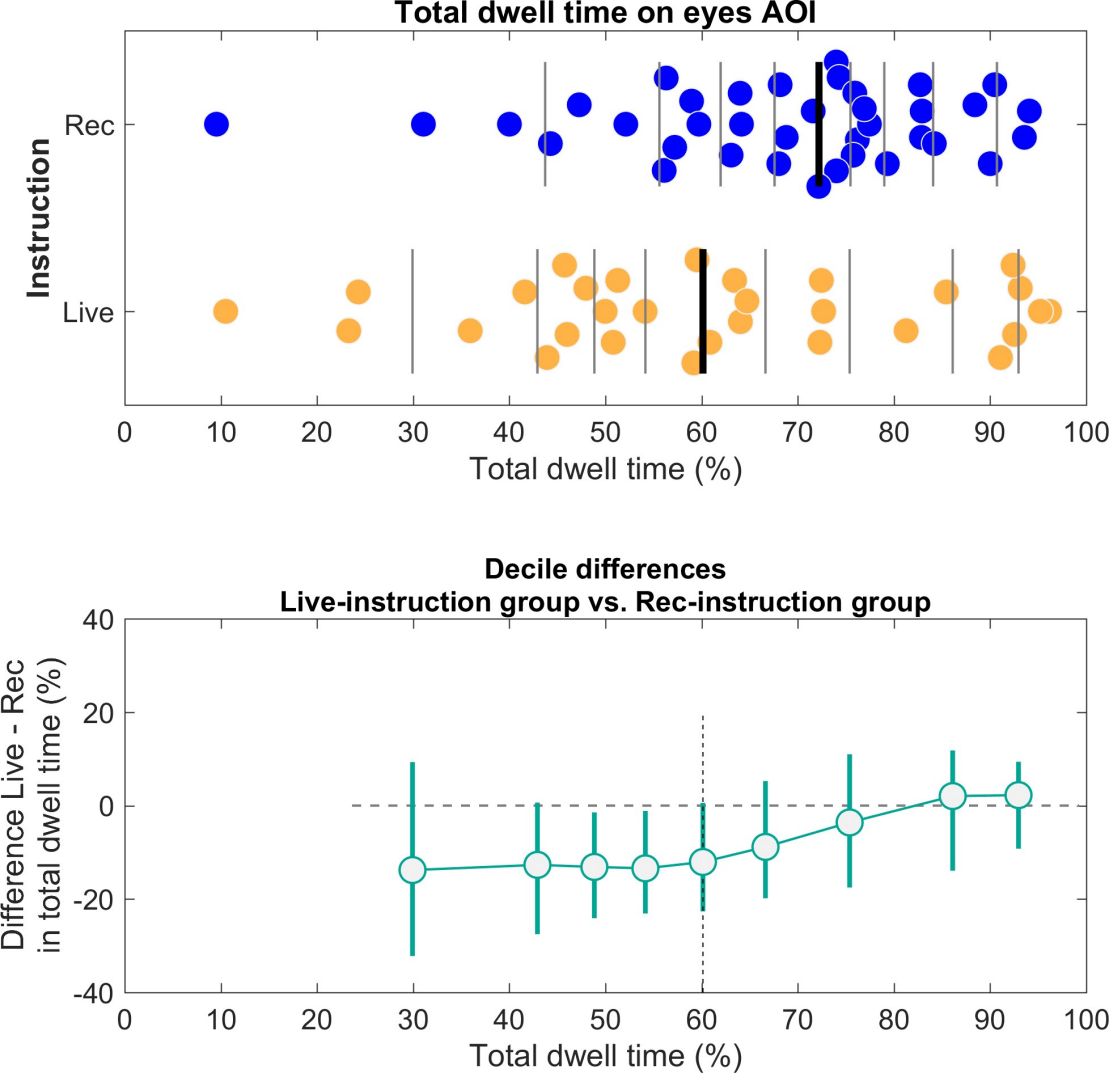
Distributions of relative total dwell time on the eyes AOI as a function of instruction-type (upper panel) and the corresponding shift function (lower panel). Upper panel: Distributions of relative total dwell time on the eyes AOI. Participants are grouped by the instruction they received prior to the experiment (live-instruction group N = 30, pre-recorded instruction group N = 38). Data points are jittered vertically to prevent overlap and each dot represents data from one participant. Vertical lines mark the deciles of the distribution. The thicker vertical line represents the median (50^th^ percentile). Lower panel: The shift function for independent groups (see [70]). The differences between deciles are plotted as a function of deciles in the live-instruction group. The horizontal dashed line represents the zero-difference line. The vertical dashed line represents the median. The x-axis shows the deciles of the live-instruction group and the y-axis shows the decile differences in relative total dwell time on the eyes AOI. The error bars of the deciles represent a 95% confidence interval computed using a percentile bootstrap (number of samples was set to 2000). The decile differences in relative total dwell time on the eyes AOI for the live-instruction group are negative for decile 1 until decile 5 (the median). However, the decile differences then become progressively less negative (decile 6 and 7) and shift back to the zero-difference line for decile 8 and 9, as indicated by the zero-difference line.

First, we compared gaze behavior at the eyes between participants who received the live-instruction and participants who received the pre-recorded-instruction. We expected that participants who received the live-instruction (N = 30) would gaze less at the eyes compared to the participants who received the pre-recorded-instruction (N = 38). In Fig 6 (upper panel), we visualized the individual data points of relative total dwell time on the eyes AOI for both groups. Then we computed the median relative total dwell time on the eyes AOI for both groups (see Fig 6, upper panel). This was done because the distribution of relative total dwell time to different areas of the face were both bounded and skewed for some AOIs. In such cases, the mean is not always a good measure to determine the central tendency of the distribution (for a discussion, see [70]). We found that the median relative total dwell time on the eyes AOI was 60.19% (Inter-Quartile-Range = 46.00% - 81.26%) for participants who received the live-instruction and 73.08% (IQR = 58.95% - 82.72%) for participants who received the pre-recorded-instruction. As is visible from Fig 6 (upper panel), many participants in both instruction-type groups looked predominantly at the eyes (approximately more than 70% of the time).

Next, we used a statistical tool called the shift function, illustrated in Fig 6 (lower panel). The shift function not only allows one to visualize and quantify whether two distributions differ, but also how they differ (for more details on the shift function, see [70]).

For example, if the relative total dwell time on the eyes AOI would be lower for participants in the live-instruction group, the decile differences should be negative and the shift function should indicate a straight line below the zero-difference line (horizontal dashed line). In our case, the shift function for independent groups indeed shows that the decile differences are negative for decile 1 until decile 5 (the median). However, the decile differences then become progressively less negative (decile 6 and 7) and shift back to the zero-difference line for decile 8 and 9. Deciles were computed using a quantile estimator (see [70] for more details) and the error bars represent a 95% confidence interval computed by using bootstrapping (number of samples was set to 2000). Finally, we computed a one-tailed Mann-Whitney-Wilcoxon test (MWW) in JASP [62] to test whether the median relative total dwell time on the eyes-AOI was significantly smaller for the live-instruction group compared with the pre-recorded instruction group. As stated, the distributions of relative total dwell times for some AOIs were skewed and bounded and we therefore used a non-parametric test. The median relative total dwell time on the eyes was not significantly smaller for the live-instruction group (*Mdn* = 60.19%) than for the pre-recorded instruction group ((*Mdn* = 73.08%), *W* = 446.0, *p* = 0.064). The effect size (here reported as a Rank-Biserial Correlation) was *r =* -0.218, which indicates a small-to-moderate negative correlation. Based on the shift-function and the effect size estimate [71], we can infer that a larger subset of the live-instruction group gazed little at the eyes of the confederates compared with the pre-recorded instruction group. Yet, a subset of the live-instruction group looked predominantly at the eyes (i.e. approximately 70–100% of the total duration of the video clip). Since we found a *p-*value of 0.064, it is important to interpret these results with some caution. It could be argued that the sample sizes of the two instruction-type groups were too small to detect significant differences in gaze behavior at the eyes on a group-level. However, in Gregory & Antolin’s study similar sample sizes were used to demonstrate a statistical difference in gaze behavior to faces between the pre-recorded-viewing group (28 participants) and the two-way-viewing group (34 participants). As such, the shift-function is particularly useful here, as it allows us to compare how the two groups differ.

#### Gaze behavior to the eyes as a function of beliefs

**Fig 7:**
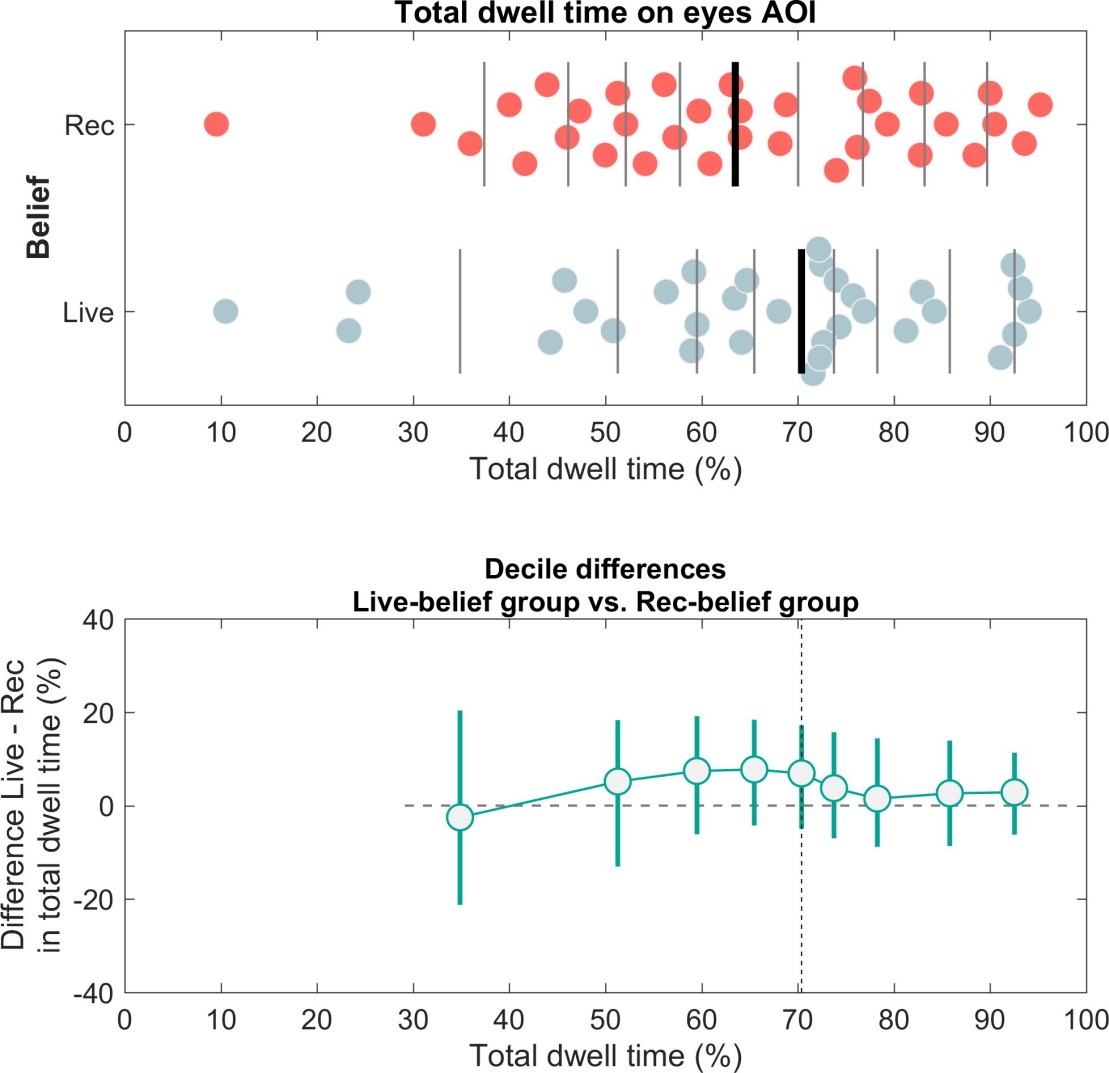
**Distributions of relative total dwell time on the eyes AOI as a function of participants self-reported ‘beliefs’ (upper panel) and the corresponding shift function (lower panel).** Upper panel: Distributions of relative total dwell time (%) on the eyes AOI. Data points are jittered vertically to prevent overlap and each dot represents data from one participant. Participants are grouped according to their ‘belief’ in whether the presentation was ‘live’ or not (live-belief group N = 33, rec-belief group N = 34). Vertical lines mark the deciles of the distribution. The thicker vertical line represents the median (50^th^ percentile). Lower panel: The shift function for independent groups. The differences between deciles are plotted as a function of deciles in the live-belief group. Decile differences in relative total dwell time are plotted on the y-axis. The horizontal dashed line represents the zero-difference line. The vertical dashed line represents the median. The x-axis shows the deciles of the live-belief group and the y-axis shows the decile differences in relative total dwell time.

As a second approach to investigate whether implying social interaction modulated gaze behavior to the eyes, we grouped participants based on the responses they gave after the experiment. We compared the relative total dwell time on the eyes of participants who answered ‘live-video connection’ (N = 33) with the relative total dwell time on the eyes AOI of participants who answered ‘pre-recorded clip’ (N = 34). In Fig 7 (upper panel), we visualized the individual data points of total dwell times to the eyes AOI for both groups. The median relative total dwell time on the eyes AOI was 72.18% (IQR = 57.63% - 82.09%) for the group of participants who answered ‘live-video connection’ and 63.53% (IQR = 49.96% - 79.31%) for the group of participants who answered ‘pre-recorded clip’. Again, we plotted the shift function to quantify how the two distributions differ (see Fig 7, lower panel). While the decile differences show a slight increase from decile 1 until decile 5, the group differences in relative total dwell time on the eyes AOI are much smaller than in the instruction-based comparison (see above). The error bars of the deciles also show consistent overlap with the zero-difference line, indicating no clear differences between the two groups.

As the median of relative total dwell time on the eyes AOI was larger for the group of participants who answered ‘live-video connection’ (*Mdn* = 72.18%) than the median of relative total dwell time on the eyes AOI for the group of participants who answered ‘pre-recorded clip’ (*Mdn* = 63.53%), a statistical analysis to verify whether participants who believed the confederate was live gazed less at the eyes would be superfluous. We conducted some follow-up tests to only look at participants whose beliefs also aligned with the instructions they received. First, we correlated the confidence ratings given by participants with regards to their responses with participants’ relative total dwell time on the eyes AOI. The correlations between confidence ratings and relative total dwell time on the eyes AOI were *r* = -0.06 for the group who answered ‘live video-connection’ and *r* = -0.11 for the group who answered ‘pre-recorded clip’. Finally, we visualized the distributions of the following two subgroups: 1) participants who received the live-instruction and who responded ‘live-video connection’ (N = 18) and 2) participants who received the pre-recorded-instruction and who responded ‘pre-recorded clip’ (N = 23), see supplementary materials. These follow-up analyses did not yield any additional insights.

#### From instruction to beliefs

**Fig 8:**
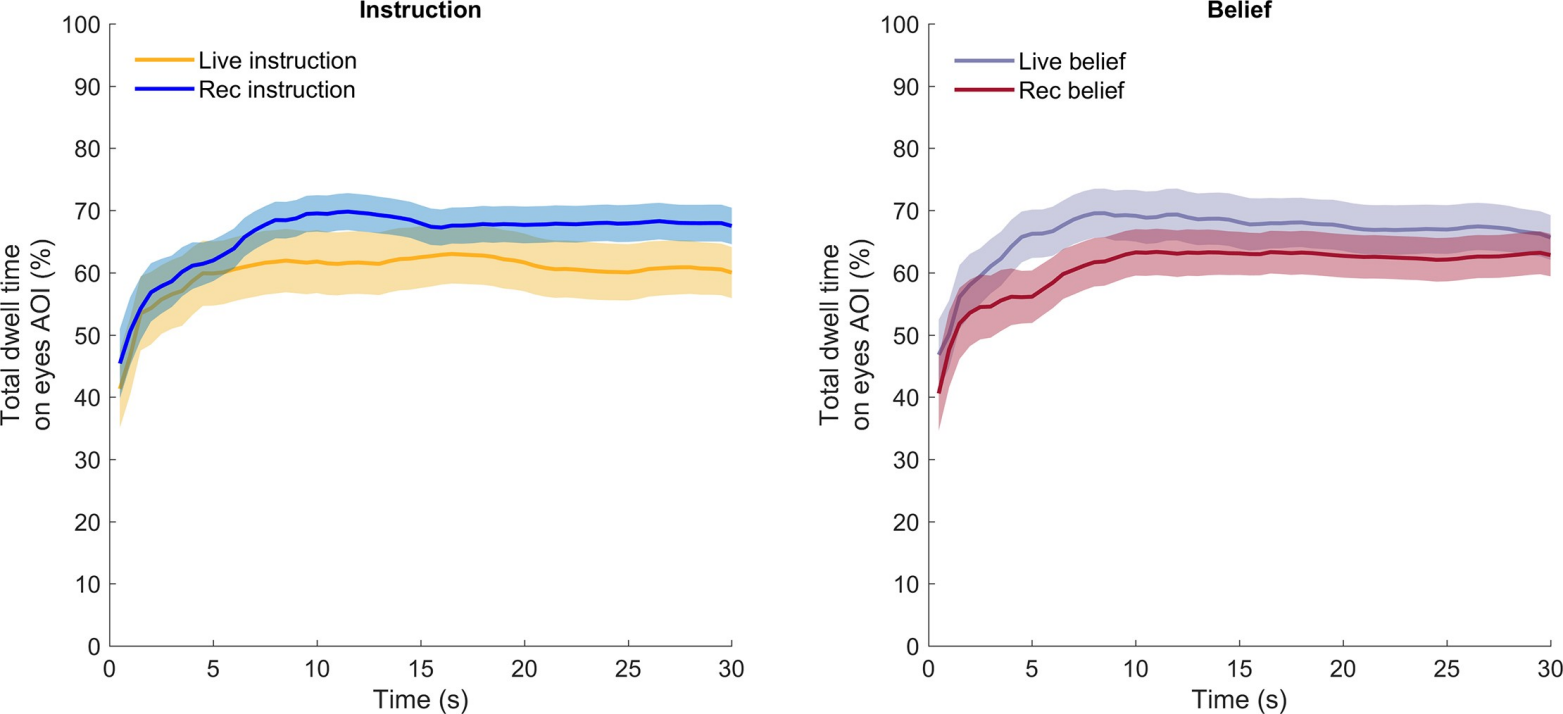
Relative total dwell time on the eyes AOI over time as a function of instruction-type (left panel) and participants’ beliefs (right panel). Lines depict average relative total dwell time (%) over time (in seconds). Shaded regions represent the standard error of the mean. The relative total dwell time on the eyes AOI is consistently lower for the live-instruction group compared with the pre-recorded instruction group. Conversely, the relative total dwell time on the eyes AOI is consistently higher for the live-belief group compared with the pre-recorded belief group.

Hitherto, we have analyzed gaze behavior to the eyes as a function of instruction-type and whether participants believed the other person was ‘live’ or not. However, one problem is that participants had to formulate their beliefs after the presentation was finished. As such, we cannot know whether participants doubted whether the confederate was ‘live’ during the presentation itself, or whether they primarily formulated post-hoc beliefs. It could be that they did not question whether the confederate was ‘live’ or not during the presentation, and if some participants did question this (as some first-person reports indicate), they only likely did so after a substantial part of the presentation. We reasoned that, if participants realized that the confederate was not ‘live’ during the presentation, we might observe changes in gaze behavior over time, which are not readily visible in aggregated measures (as in Figs 6 and 7). To check this, we visualized relative total dwell time on the eyes AOI over the course of the presentation. As is visible from Fig 8, the temporal development of total dwell time on the eyes AOI seems to be representative of the aggregated endpoints. Relative total dwell time on the eyes AOI is consistently lower for the live-instruction group compared with the pre-recorded instruction group Conversely, relative total dwell time on the eyes AOI is consistently higher for the live-belief group compared with the pre-recorded belief group. Note that there is considerable overlap between the two groups, as is visible from the shaded regions. All in all, we do not observe clear changes in gaze behavior to the eyes over the course of the presentation. The initial increase in relative total dwell time on the eyes AOI at the beginning of the presentation (approximately the first 5 seconds) is likely due to different fixation positions at the beginning of the recording.

## Discussion

We investigated why people experience the potential for social interaction and tested whether implying social interaction modulated gaze behavior to faces, specifically the eyes. Previous work has shown that people look differently at ‘live’ people compared with videos of faces [11], or when they believe that they are being watched by others [45]. However, these studies have primarily investigated how social presence, in the absence of social interaction, influences gaze behavior to faces. Our study was designed to overtly imply the potential for social interaction with another person and simultaneously measure how people look at faces. First, we will discuss our findings regarding participants’ experience of social interaction and then we will discuss our findings with regard to our hypotheses about gaze behavior to faces and eyes.

### Implying social interaction: The importance of responsiveness and synchronicity

Our experiment was a novel attempt at implying the potential for social interaction. In previous work, potential for social interaction was only minimally implied, as confederates did not convey the initiative to engage in interaction (cf. [11, 45]). Our goal was to uncover why social interaction may be experienced and how gaze behavior to the eyes was modulated as a result. Our research setting was structured in such a way that participants were be led to believe that they would engage in social interaction with another person. Indeed, our paradigm seemed to have succeeded in conveying the potential for social interaction, as many people responded that they felt that the other person was ‘interacting’ and ‘responding’, even though participants were all presented with a pre-recorded video clip. Based on participants’ responses, half of the participants believed that they were presented with a person through a live-video connection. Participants’ responses were not strongly correlated with instruction-type or video-type, although some participants did base their judgements on the instruction they received prior to the experiment. Note that we only asked participants to judge whether they believed the other person was ‘live’ after the experiment. We found that many participants, when judging whether the confederate was ‘live’ or not, commented on the temporal and reciprocal aspects of the confederate’s behavior (e.g. timing of the gaze cues and facial expressions). Interestingly, while some participants reported that they perceived the confederate’s behavior to be ‘interactive’ (e.g. synchronicity with their own behavior), other participants perceived the confederate’s reactions to be strange and discontinuous. This clearly shows the importance of the subtle temporal interplay between the participants’ behavior and the perceived reciprocity and responsiveness of the confederate. As such, these first-person reports provide important insights regarding participants’ subjective experience of potential social interaction, and also to what extent potential interactions can be simulated in a non-interactive experimental setting.

Our first-person reports may be particularly relevant for researchers studying social cognition and gaze behavior during social interactions [10]. In particular, the insights we derived from the first-person reports could be a starting point for the development of experimental designs aimed to simulate the experience of social interaction [25, 26, 72]. First-person reports indicate that the temporal and reciprocal aspects of the confederate’s behavior were particularly important for how people experienced the potential for interaction. We wondered how one could further disentangle or model these aspects in order to optimize the experience of social interactions in experimental settings. One clever approach to disentangle these factors was achieved by Pfeiffer et al., [73]. In their so-called ‘non-verbal Turing test’, participants were asked to judge whether the behavior of a virtual avatar was controlled by a person or by a computer program. The ingenuity of this study was that the gaze direction of the virtual avatar was responsive to where the participant was looking. By systematically varying between gaze-following and gaze-aversion, Pfeiffer et al. [73] demonstrated that participants’ judgement about the ‘humanness’ of the virtual avatar was dependent on the gaze congruency (i.e. gaze direction relative to person’s gaze) and gaze contingency (i.e. the temporal dynamics). Furthermore, judgements about ‘humanness’ were also dependent on participants’ beliefs about whether the virtual avatar would behave competitively or cooperatively. In other words, the simulated responsiveness of the virtual avatar influenced participants’ sense of social interaction. Brandi et al. [74] also used a virtual avatar whose gaze behavior was responsive to the measured gaze behavior of the participant. Brandi and colleagues found that systematic changes in the responsiveness of the virtual avatar also influenced participants’ judgements about the ‘humanness’ and social agency of the virtual avatar.

Taken together, implying social interaction certainly seems possible. However, staging a full-fledged experience of social interaction may not be as straightforward as merely implying social presence. In our study, the first-person responses indicated that many participants perceived the confederate as being responsive to their own behavior. Yet, many participants also perceived the confederate’s behavior as being ‘out of sync’. While social presence and potential interactions may overlap in certain situations, social interaction likely constitutes a qualitatively different domain of the social context. The potential for social interaction likely depends on whether potential interactors overtly signal and engage in ‘interactive’ behavior (see also [37, 75]). Importantly, we only used a limited set of possible social gaze cues and obviously excluded many social signals such as speech and gestures that are also typically used by people to interact [20]. Furthermore, we primarily focused on why people experienced interaction with another person and whether implying social interaction modulated gaze behavior to the eyes.

### Implying social interaction and its influence on gaze behavior to the eyes

Previous studies have demonstrated that the actual, imagined or implied presence of others modulated basic processes of visual attention and gaze behavior, for example, how people look at other people’s faces [11, 39, 45]. However, few studies have specifically addressed whether implying social interaction modulates gaze behavior to the eyes. Based on previous studies, we expected that prolonged direct gaze with a stranger would cause people to avoid looking at that person’s eyes (cf. [11, 45]. The ‘social risk’ hypothesis, for example, predicts that prolonged direct gaze may result in overall gaze avoidance [45], as prolonged direct gaze with a stranger is typically regarded as inappropriate, uncanny, or may even be perceived as aggressive and threatening [41, 46–48]. In our study, we expected that participants who believed they were being looked at by a ‘live’ confederate would gaze less at the eyes of that person. However, our data only partly supports this hypothesis. We found that a relatively larger subset of the live-instruction group gazed little at the eyes of the confederate compared with the pre-recorded-instruction group. However, many participants from the live-instruction group predominantly gazed at the eyes of the confederate.

In sum, we found only small differences in gaze behavior to the eyes between the live-instruction group and the pre-recorded-instruction group (as indicated by the shift function). Furthermore, when grouping participants based on their self-reported beliefs, the median relative total dwell time on the eyes AOI was numerically higher for the live-belief group participants compared with the pre-recorded belief group. This was against our initial expectations. However, as clarified by the first-person responses, participants who received the live-instruction were not necessarily the same participants who believed that the confederate was ‘live’, making this result harder to interpret. Moreover, confidence ratings did not correlate with participants’ relative total dwell time on the eyes AOI, nor were they associated with instruction-type or video clip-type.

One could argue that our study does not have a large enough sample size. Yet, Gregory & Antolin’s study [45] used a similar number of participants and reported much larger effect sizes, as well as significant statistical differences between groups based on instruction-type. Although the study by Gregory & Antolin probably offers the best comparison for sample sizes given the overlap in research questions and hypotheses about gaze behavior to eyes and faces, the experimental procedures and analyses also differ on several levels. For example, the confederate in the video clip in Gregory & Antolin’s study did not generally look at the participants, but only did so for a brief moment. In our study, all video clips displayed confederates who directly looked at the observer (into the camera), which likely constitutes a rather different viewing experience. Importantly, we also used different methodological tools to map gaze behavior unto dynamic face stimuli. This allowed us to measure gaze behavior to specific facial features (e.g. eyes, nose, mouth), whereas Gregory & Antolin’s study focused primarily on gaze behavior to the face in comparison with the background of the scene. For two smaller groups of participants, they report gaze behavior to the eyes. In our study, we focused on the entire distributions of relative total dwell time on the eyes AOI, and showed that participants varied greatly in how long they gazed at the eyes of the confederate, irrespective of the instruction-type, video clip-type or their self-reported belief. Indeed, other eye-tracking studies have reported large inter-individual differences in face scanning patterns. For example, several eye-tracking studies have indicated that how individual observers look at faces can be characterized by idiosyncratic face scanning patterns that are stable over time (see e.g. [66, 67, 69]. Simply put, some people may preferentially fixate the eyes while others generally fixate the nose or the mouth area. Furthermore, differences in gaze behavior to faces also seem to be modulated by specific task-demands, for example, during speaking, listening, free viewing, face recognition and emotion recognition, but also by cultural factors [76]. We did not specifically design this study in order to investigate to what extent our participants exhibited idiosyncratic biases in gaze behavior to faces, nor did we impose a specific task to participants. However, the extent to which gaze behavior to the eyes was modulated as a function of instruction-type could have been outweighed by these inter-individual differences.

## Conclusions

We demonstrated a novel method for implying the potential for social interaction with another person. We have highlighted several factors that are likely to be important for implying social interaction, namely the behavior of the confederate, the attitudes of participants, and various contextual and technical aspects of the research setting. Furthermore, we found only marginal evidence supporting the ‘social risk’ hypothesis. In fact, participants varied greatly in gaze behavior to the eyes, irrespective of instruction-type or participants’ beliefs about the possibility to interact. Based on these results, as well as insights from previous research, we think that specific factors of the social context likely modulate how people allocate their gaze. Furthermore, we think that individual differences in gaze behavior to faces need to be more explicitly considered in future experiments, as they could be crucial to our understanding of how gaze behavior is allocated across different social contexts.

## Supporting information

S1 Fig Follow-up analysis of gaze behavior to eyes for subgroups (instruction-type aligned with participants’ beliefs). (DOCX)

S2 Fig (TIF)
