# Supplementary material for: Implying social interaction and its influence on gaze behavior to the eyes
Source: PLoS One. 2020 Feb 24;15(2):e0229203. doi: 10.1371/journal.pone.0229203 (PMC7039466; doi:10.1371/journal.pone.0229203)
Supplement: S1 Fig — (DOCX) [file pone.0229203.s001.docx]

**S1 Fig**. **Follow-up analysis of gaze behavior to the eyes for subgroups (instruction-type aligned with participants’ beliefs).**

Distributions of relative total dwell times on the eyes AOI for two subgroups: instructions aligned with beliefs (lower panel). Upper panel: Distributions of relative total dwell time on the eyes AOI. Participants are grouped by the instruction they received aligned with their self-reported beliefs after the experiment. Live-group shows data points from participants who received the live-instruction and answered ‘live video-connection’ (N=18). Rec-group shows data points from participants who received the pre-recorded clip instruction and answered ‘pre-recorded clip’ (N=23). Data points are jittered vertically to prevent overlap and each dot represents data from one participant. Vertical lines mark the deciles of the distribution. The thicker vertical line represents the median (50^th^ percentile). Lower panel: The shift function for independent groups. The differences between deciles are plotted as a function of deciles in the live- group. The horizontal dashed line represent the zero-difference line. The vertical dashed line represents the median. The x-axis shows the deciles of the live-group and the y-axis shows the decile differences in total dwell time. The error bars of the deciles represent a 95% confidence interval which was computed by using a percentile bootstrap (number of samples was set to 2000).
