# Supplementary figures and images for: Implying social interaction and its influence on gaze behavior to the eyes
Source: PLoS One. 2020 Feb 24;15(2):e0229203. doi: 10.1371/journal.pone.0229203 (PMC7039466; doi:10.1371/journal.pone.0229203)

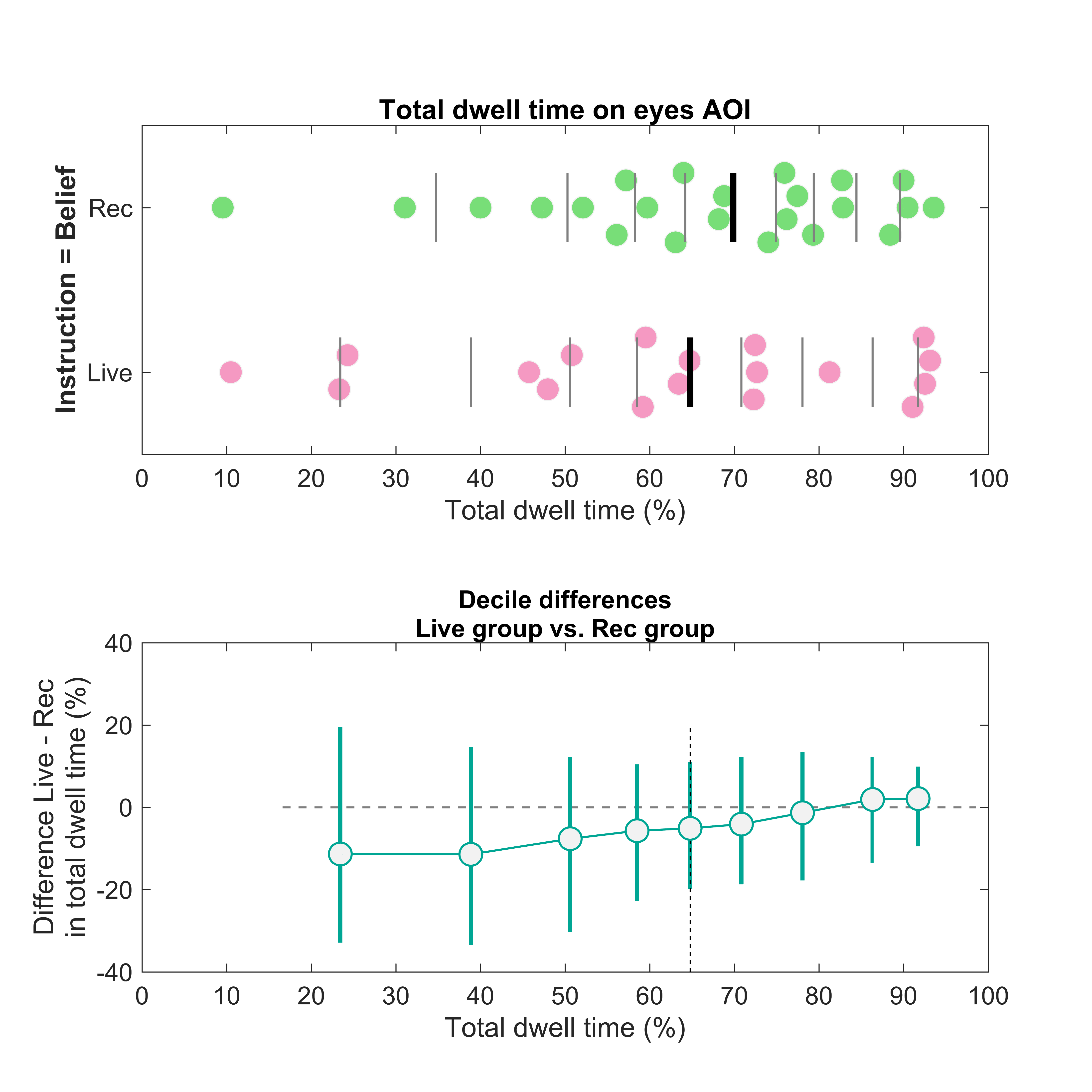

Supplement: S2 Fig — (TIF) [file pone.0229203.s002.tif]
